# Acute Endotoxemia-Induced Respiratory and Intestinal Dysbiosis

**DOI:** 10.3390/ijms231911602

**Published:** 2022-10-01

**Authors:** Evy Goossens, Jianhui Li, Chana Callens, Nathalie Van Rysselberghe, Hannele Kettunen, Juhani Vuorenmaa, Natalia Garcia Gonzalez, Claude Libert, Richard Ducatelle, Filip Van Immerseel

**Affiliations:** 1 Livestock Gut Health Team (LiGHT) Ghent, Department of Pathobiology, Pharmacology and Zoological Medicine, Faculty of Veterinary Medicine, Ghent University, Salisburylaan 133, 9820 Merelbeke, Belgium; 2 Department of Livestock Production, College of Animal Science, Shanxi Agricultural University, Jinzhong 030801, China; 3 Hankkija Oy, Peltokuumolantie 4, FI-05801 Hyvinkää, Finland; 4 Department for Molecular Biomedical Research, Vlaams Instituut voor Biotechnologie (VIB), 9052 Ghent, Belgium

**Keywords:** sepsis, systemic inflammatory response syndrome, acute lung injury, LPS, microbiota, dysbiosis, inflammation

## Abstract

Systemic inflammatory response syndrome (SIRS) is a severe condition characterized by systemic inflammation, which may lead to multiple organ failure, shock and death. SIRS is common in burn patients, pancreatitis and sepsis. SIRS is often accompanied by intestinal dysbiosis. However, the mechanism, role and details of microbiome alterations during the early phase of acute SIRS are not completely understood. The current study aimed to characterize the dynamic alterations of both the intestinal and respiratory microbiome at two timepoints during the early phase of acute SIRS (4 and 8 h after LPS) and link these to the host response in a mouse model of a LPS-induced lethal SIRS. Acute SIRS had no effect on the microbiome in the large intestine but induced a rapid dysbiosis in the small intestine, which resembled the microbiome alterations commonly observed in SIRS patients. Later in the disease progression, a dysbiosis of the respiratory microbiome was observed, which was associated with the MMP9 expression in the lungs. Although similar bacteria were increased in both the lung and the small intestine, no evidence for a gut-lung translocation was observed. Gut dysbiosis is commonly observed in diseases involving inflammation in the gut. However, whether the inflammatory response associated with SIRS and sepsis can directly cause gut dysbiosis was still unclear. In the current study we provide evidence that a LPS-induced SIRS can directly cause dysbiosis of the small intestinal and respiratory microbiome.

## 1. Introduction

Systemic inflammatory response syndrome (SIRS) is a serious condition that usually results in multiple organ dysfunctions. One of the most common and earliest affected organs in SIRS are the lungs, resulting in an acute lung injury (ALI) or acute respiratory distress syndrome (ARDS) [1,2]. SIRS may occur as a consequence of acute inflammation caused by either an infection leading to sepsis, or in the absence of an infection, e.g., as a result of trauma, bleeding, pancreatitis or burns [3]. Endotoxemia is often used to study the acute inflammatory response associated with early sepsis [4,5]. Endotoxemia is induced using endotoxins, a.k.a. lipopolysaccharide (LPS), one of the principal components of the outer membrane of Gram-negative bacteria. The LPS binds to the Toll-like receptor (TLR) 4, which is expressed on many cell types, and signals resulting in the activation of several kinases and transcription factors, such as NF-κB, jun, fos, IRFs and the mTOR/STAT3 pathway, which lead to the transcription of numerous genes, including genes encoding the production of cytokines such as IL1β, IL6 and TNFα, amongst others [5,6]. The increased cytokine levels may cause damage to the respiratory and gut epithelium, leading to impaired barriers, bacteremia and sepsis [2,6,7]. Matrix metalloproteinases (MMPs) function as key regulators of this hyperinflammation, both through the degradation of the extracellular matrix, which contributes to the epithelial dysfunction as well as through the processing of chemokines, cytokines and cell surface receptors, thereby directly influencing the inflammatory cascade [8,9,10,11,12,13].

Various animal models are used to study SIRS, ranging from “metabolic endotoxemia” induced by chronic exposure to low levels of LPS [14,15], to “acute, lethal endotoxic shock” resulting from challenge with a high dose of LPS [11,16,17,18,19]. Using these models, the role of the MMPs and the inflammatory cascade associated with various stages and degrees of SIRS has been the topic of intensive research [6,20,21,22,23]. SIRS induced by LPS causes barrier problems and inflammation in numerous organs, such as the intestinal epithelium, the lungs (causing acute lung injury (ALI), a form of acute respiratory distress syndrome (ARDS)), liver and kidney (leading to acute kidney injury (AKI)), as well as the brain. Recently, evidence has pointed towards an important role of the microbiome in SIRS, sepsis and ARDS [24,25,26]. Indeed, dysbiosis of the intestinal microbiome has been identified as an important predisposing (risk) factor for sepsis [27,28]. However, after the onset of sepsis alterations in the gut microbiome, it can worsen the disease progression and induce multiple organ failure. The causal mechanisms behind the gut microbiome disruption after the onset of sepsis are not completely understood. A link with therapeutic interventions such as antibiotic treatment, opioids or parenteral nutrition has previously been established [29,30]. Whether the inflammatory response associated with SIRS and sepsis can directly cause gut dysbiosis is still unclear. Therefore, the aim of the current study was to investigate the respiratory and intestinal microbiota changes in response to acute SIRS and link this to the host response in a mouse model of a LPS-induced lethal SIRS.

## 2. Results

### 2.1. LPS-Induced Acute SIRS with Acute Lung Injury

To characterize how the respiratory and gut microbiome respond to acute inflammation, mice were injected with a lethal dose of LPS. This LPS-induced systemic inflammation is a well-accepted and validated model for SIRS, characterized by the pathological changes that strongly resemble sepsis, and is lethal within 48 h to 72 h after the challenge [11,31,32,33].

**Figure 1:**
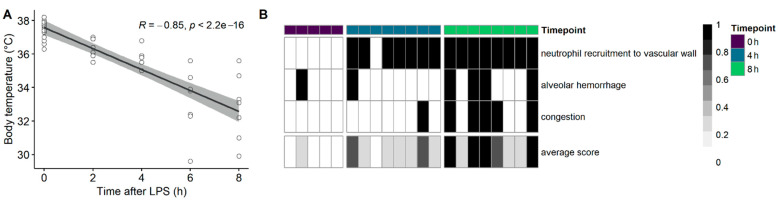
Intraperitoneal LPS challenge results in the acute drop in body temperature, with an acute lung injury. C57BL/6J mice were injected intraperitoneally with a lethal dose of LPS, and the body temperature was monitored over time (**A**). Mice were sacrificed before the LPS injection (0 h, healthy mice), at 4 or 8 h after the LPS injection. (**B**) Histopathological assessment of the lungs for the presence of the neutrophil recruitment to the vascular wall, an alveolar congestion or hemorrhage. The bottom row represents the average lesion score per lung. Each column represents a single mouse, sampled either before the LPS challenge (purple), 4 h after the LPS (blue) or 8 h after the LPS (green).

**Figure A1:**
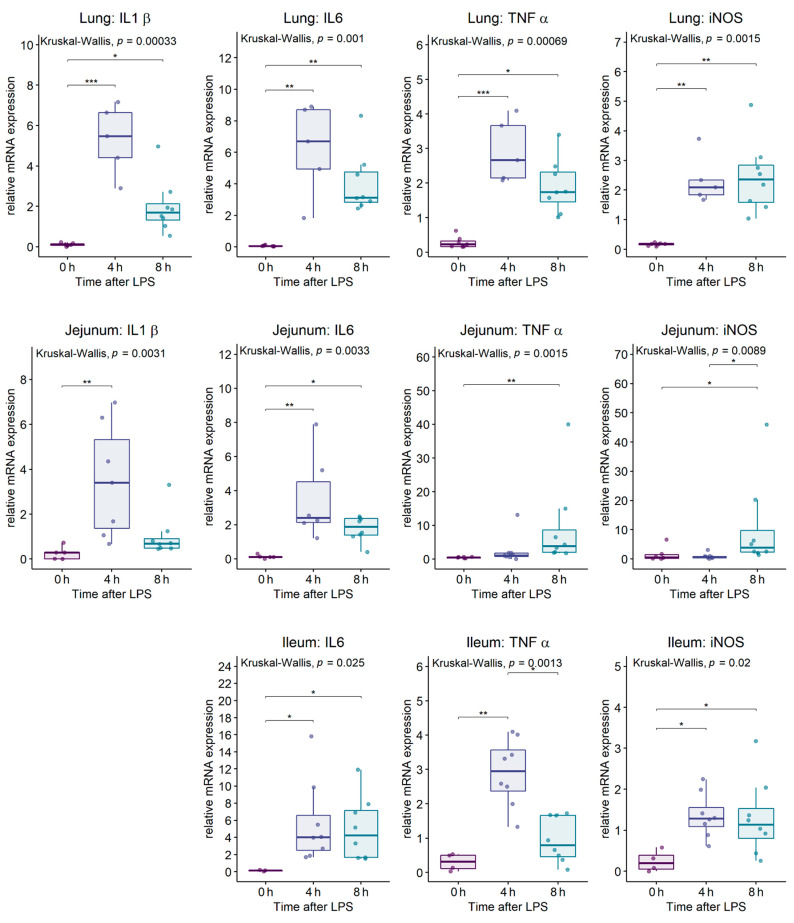
Relative inflammatory gene expression levels in the lung (**top** row), jejunum (**middle** row) or ileum (**bottom** row) at 0 h (healthy mice), 4 h or 8 h after the IP LPS challenge. Significant differences between the timepoints after LPS are highlighted with: * *p* < 0.05, ** *p* < 0.01 or *** *p* < 0.001.

An intraperitoneal (IP) LPS challenge resulted in a significant drop in body temperature (*p* < 0.0001; Figure 1A), together with a pro-inflammatory cytokine gene expression (Figure A1), indicating that the challenge induced an acute SIRS. Evidence of acute lung injury was indicated by the migration of neutrophils to the alveolar walls, alveolar congestion and hemorrhages (Kruskall-Wallis on the average lung histopathological lesion score: *p* = 0.0013; 0 h vs. 4 h: *p* = 0.1908; 0 h vs. 8 h: *p* = 0.0028; Figure 1B). These macroscopic changes were accompanied by an acute inflammatory response, as observed by the induction of genes encoding for iNOS (inducible nitric oxide synthase), IL1β, IL6 and TNFα in the lung (Figure A1).

### 2.2. Effect of the Intraperitoneal LPS Challenge on the Respiratory and Intestinal Microbial Diversity

To determine the effect of a LPS-induced SIRS and ALI/ARDS on the overall intestinal and respiratory microbial diversity, the microbial richness, diversity and community structures of both the intestinal and respiratory samples from unchallenged healthy mice were compared to samples from mice at 4 h or 8 h after the LPS challenge.

The microbial complexity in the lungs or the intestine was assessed by calculating the estimated ASV richness (Chao1) or the estimated community diversity (Shannon index) in each sample. The intraperitoneal LPS injection had no effect on the microbial alpha diversity in the colon (Table 1). However, when comparing the microbial complexity at 4 h and 8 h after the LPS, a significant increase in the microbial richness (Chao1) in the lungs was observed, which was mainly due to a low richness at 4 h after the LPS. Furthermore, a decrease in the ileal microbial diversity (Shannon) was observed at 8 h after the LPS challenge (Table 1).

**Figure 2:**
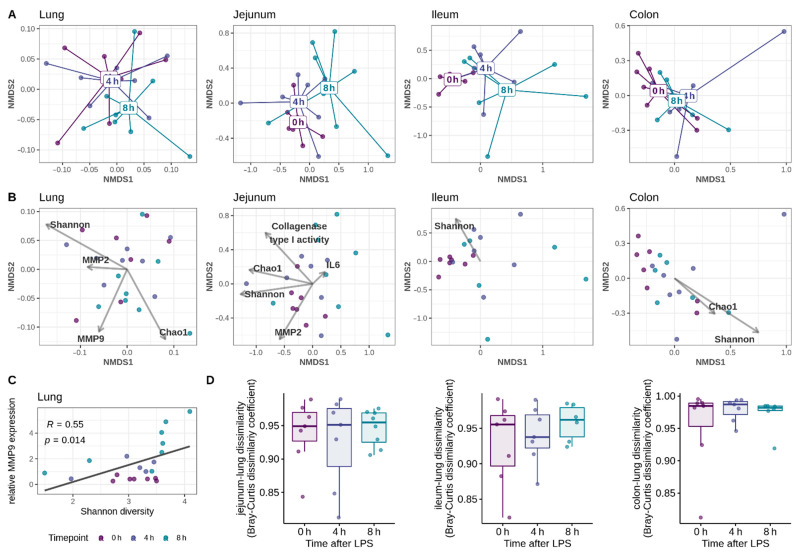
NMDS plot of the respiratory and intestinal microbiota following the intraperitoneal LPS injection. C57BL/6J mice were injected intraperitoneally with a lethal dose of LPS. Mice were sacrificed before the LPS injection (0 h, healthy mice), at 4 or 8 h after the LPS injection. (**A**,**B**). Nonmetric multidimensional scaling (NMDS) plot of the Bray–Curtis dissimilarities. Lung and intestinal samples were collected from the unchallenged mice (0 h), 4 h, or 8 h after the LPS challenge. Each point represents a single mouse microbiome. (**A**) In the top row, the ‘spider webs’ link each sample to the centroid of the respective timepoint after the LPS challenge. (**B**) In the middle row, the variables significantly associated with the beta diversity are added (significance determined using PERMANOVA). The arrow points towards the direction of the most rapid change in the variable, the arrow length is proportional to the correlations between the variable and the ordination. (**C**) The microbial Shannon diversity in the lungs was positively correlated to the MMP9 expression in the lung tissue (Spearman rank correlation). (**D**) Gut-lung community similarity in healthy (0 h) mice, or at 4 h or 8 h after the LPS challenge. For each mouse, the community dissimilarity was calculated for the paired gut-lung communities, using the Bray–Curtis dissimilarity metric. A dissimilarity value of 1 means no species are shared between the gut and lung microbiome, whereas 0 indicates the gut and lung have the same composition.

The Bray–Curtis dissimilarity was used to investigate the effect of the IP LPS injection on the beta diversity of the respiratory (lung) or intestinal (jejunum, ileum, colon) microbiota (Figure 2A). The LPS challenge had no major effect on the overall microbial community composition in the lungs (Table 2). At 4h after the LPS injection, the respiratory community structure could not be discriminated from the unchallenged mice (0 h) (Figure 2A and Table A1). However, at 8 h after the LPS injection, the overall community composition in the lungs was significantly different from the respiratory community structure at 4 h after the LPS (Table A1). In the small intestine, a clear separation of the samples based on the time after the LPS challenge was observed (Figure 2A). Indeed, the LPS challenge increased the variation between the small intestinal microbial communities of the different individuals (significant increase in dispersion), together with an alteration of the overall microbial community composition (Table 2 and Table A1). More specifically, the LPS challenge accounted for 15.49% (jejunal microbiome) to 24.09% (ileal microbiome) of the variation between the microbiota samples. No effect of the LPS challenge on the colonic microbial community structure was observed (Figure 2A and Table 2).

### 2.3. Intraperitoneal LPS Challenge Induces a Dysbiosis of the Small Intestinal and Respiratory Microbiome, Which Is Not Linked to a Gut-Lung Bacterial Translocation

**Figure 3:**
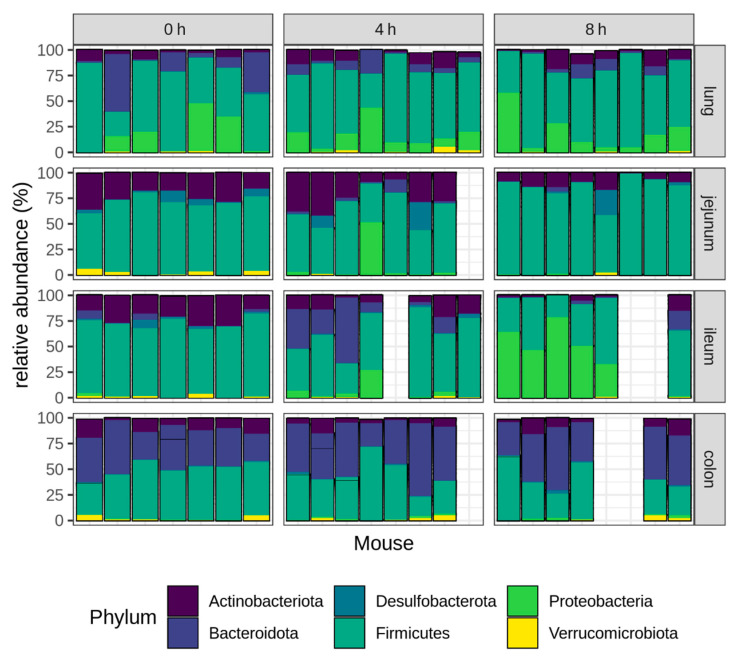
Relative abundance (%) of the six most abundant phyla in the lung, jejunum, ileum or colon at different timepoints after the intraperitoneal LPS injection. Lung and intestinal samples were collected from the unchallenged mice (0 h), as well as 4 h or 8 h after the LPS challenge. Each bar represents an individual mouse microbiome. For seven samples, the 16S sequencing failed, resulting in empty bars on the graph.

As the LPS challenge resulted in a significant shift in the microbial community structure, we further focused on the taxonomic composition of the respiratory and intestinal microbiome, and how this is affected by the LPS challenge. The Firmicutes was the main phylum detected in the lungs of the unchallenged, healthy mice (57.82%), followed by the phyla Bacteroidota (18.91%), Proteobacteria (16.99%) and Actinobacteriota (5.09%) (Figure 3). The small intestine of healthy mice was also dominated by members of the Firmicutes (Jejunum: 69.01%; Ileum: 71.45%) and followed by the Actinobacteriota (Jejunum: 23.44%; Ileum: 21.58%). The main phyla observed in the colon of healthy mice were the Firmicutes (47.64%), Bacteroidota (37.99%) and Actinobacteriota (10.34%).

The intraperitoneal LPS challenge had no effect on the distribution of the bacterial phyla in the colon, but resulted in a significant shift in both the small intestinal and respiratory microbiome, with the most pronounced LPS effect observed in the lungs and the ileum (Figure 3).

In the lungs, the LPS challenge resulted in a reduction of the Bacteroidota, shifting from 18.9% in the unchallenged mice to respectively 8.09% and 5.29% in the lungs at 4 h and 8 h after the LPS injection (0 h vs. 4 h: *p* = 0.0006; 0 h vs. 8 h: *p* = 0.0002).

The main LPS effect in the jejunum was observed for the phylum Actinobacteriota, which decreased from 23.44% in the unchallenged mice to 9.78% at 8 h after the LPS (*p* = 0.015). Additionally, an increase in the phyla Bacteroidota (0 h vs. 4 h: *p* < 0.001; 0 h vs. 8 h: *p* = 0.007), Firmicutes (0 h vs. 8 h: *p* = 0.003) and Proteobacteria (0 h vs. 4 h: *p* < 0.001; 0 h vs. 8 h: *p* = 0.003), together with a decrease in the Verrucomicrobiota (0 h vs. 4 h: *p* = 0.031) was observed in the jejunum (Figure 3).

The ileal microbiome was characterized by an expansion of the Proteobacteria from 0.44% in the unchallenged mice to 6.37% at 4 h after the LPS, and 45.7% at 8 h after the LPS challenge (0 h vs. 4 h: *p* = 0.010; 0 h vs. 8 h: *p* < 0.001). This bloom of the Proteobacteria is a well-known hallmark of dysbiosis. Furthermore, the phylum Bacteroidota was increased at 4 h but not at 8 h after the LPS (0 h: 2.42%—4 h: 22.15%—8 h: 4.04%; 0 h vs. 4 h: *p* < 0.001; 0 h vs. 8 h: *p* = 0.207), and the Actinobacteriota tended to be increased at 8 h after the LPS (0 h: 21.58%—8 h: 4.09%; *p* = 0.070) (Figure 3).

At the family level, the LPS challenge resulted in a significant increase of the families *Bacillaceae*, *Pasteurellaceae* and *Streptococcaceae* in the lungs (Table 3). This increase in the *Pasteurellaceae* and to a lesser extent also in the *Streptococcaceae,* could also be observed in the ileum, but not the jejunum of the LPS-challenged mice. In both the lungs and the ileum, the increase in *Streptococcaceae* was entirely due to the genus *Streptococcus*, whereas the increase in *Pasteurellaceae* could be attributed to an increase in the genus *Muribacter* (Table A2). In the small intestine (both jejunum and ileum), the LPS challenge resulted in a marked increase of the families *Enterococcaceae* (genus *Enterococcus*) and *Enterobacteriaceae* (genus *Escherichia-Shigella*). Furthermore, the IP LPS injection shifted the ileal microbiome towards an increase in the families *Clostridiaceae* (mainly genus *Candidatus Arthromitus*), *Burkholderiaceae* (genus *Ralstonia*) and two families belonging to the order Rhizobiales, whereas the ileal families *Eggerthellaceae* and *Christensellaceae* were the only families that were reduced after the LPS challenge (Table 3 and Table A2). In addition, multiple low abundant families were increased due to the LPS challenge in both the small and large intestine (Table 3). These results strongly suggest that the LPS-induced SIRS and ALI/ARDS are associated with a dysbiosis of the respiratory and small intestinal microbiota, as highlighted by significant shifts in the bacterial populations from a broad range of taxonomic groups.

As the lungs of the LPS-challenged mice were increased in bacterial taxa that were also increased in the ileum, this might point towards a LPS-induced leaky gut and the concomitant bacterial translocation from the gut to the respiratory system. To determine if the post-LPS lung communities resembled those of the gut, the bacterial community membership of the gut and the lung communities within each mouse were compared (Figure 2D). Both in the healthy mice and after the LPS-challenge, only a small similarity between the overall community composition of the lung and the gut was observed, and the LPS-challenge had no effect on the gut-lung dissimilarity score. Additional calculations of the percentage of the shared species (ASVs) between the gut and lung communities showed similar results (non-identical ASVs in the paired gut-lung microbiomes; data not shown), indicating that the LPS-induced microbial shifts in the lungs are not linked to an increase in bacteria originating from the gut.

### 2.4. Factors Related to the Respiratory and Intestinal Microbiome Community Structure

To determine whether the shifts in the microbial community structure could be linked to a change in the alpha diversity, a PERMANOVA analysis was performed to associate the beta diversity measurements with either the bacterial richness (Chao1) or diversity (Shannon). In both the lungs, the small intestine and the colon, the alpha diversity metrics were significantly linked with the community structure (Table 4). This association was visualized using the NMDS ordination biplots, which indicated a correlation between the bacterial richness (Chao1) and diversity (Shannon) in both the jejunum and the colon, an observation that was confirmed by the Spearman correlation analysis (R > 0.5; *p* < 0.015 for all intestinal segments). In the lungs, however, the NMDS biplots showed that the Shannon diversity and the Chao1 richness had an opposite effect on the bacterial community structure (Figure 2B). Furthermore, the Chao1 richness was not correlated to the Shannon diversity (R = 0.15; *p* = 0.48), indicating that an increase in the bacterial richness in the lungs was not linked to an increase in diversity.

**Figure A2:**
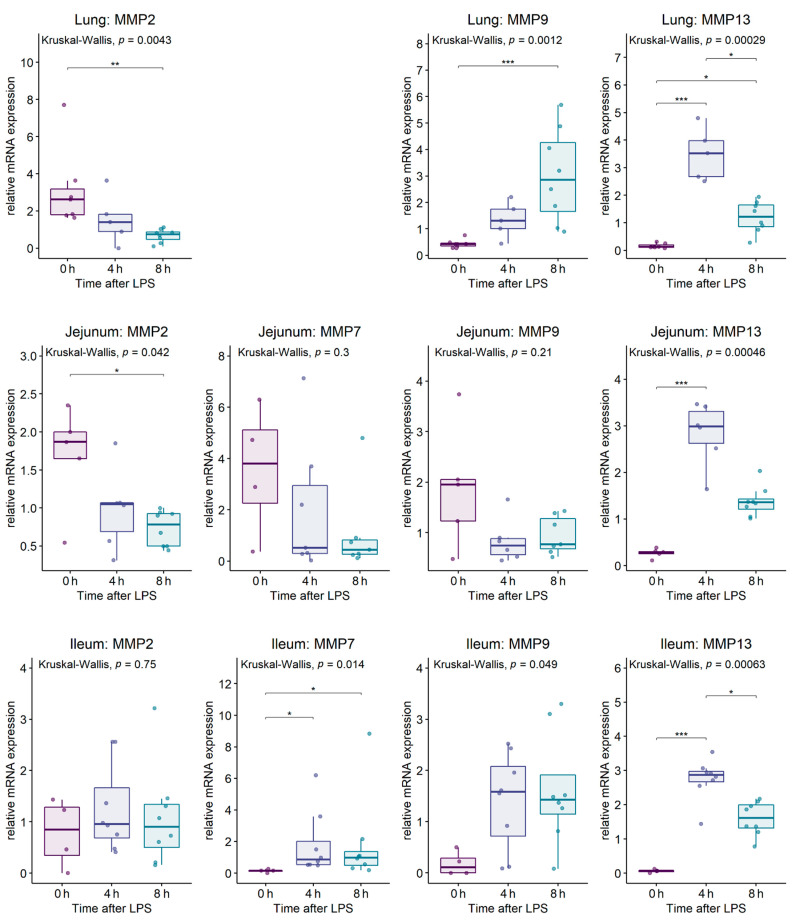
Relative expression levels of the selected matrix metalloproteinase (MMP) genes in the lung (**top** row), jejunum (**middle** row) or ileum (**bottom** row) at 0 h (healthy mice), 4 h or 8 h after the IP LPS challenge. Significant differences between the groups are highlighted with: * *p* < 0.05, ** *p* < 0.01 or *** *p* < 0.001.

**Figure A3:**
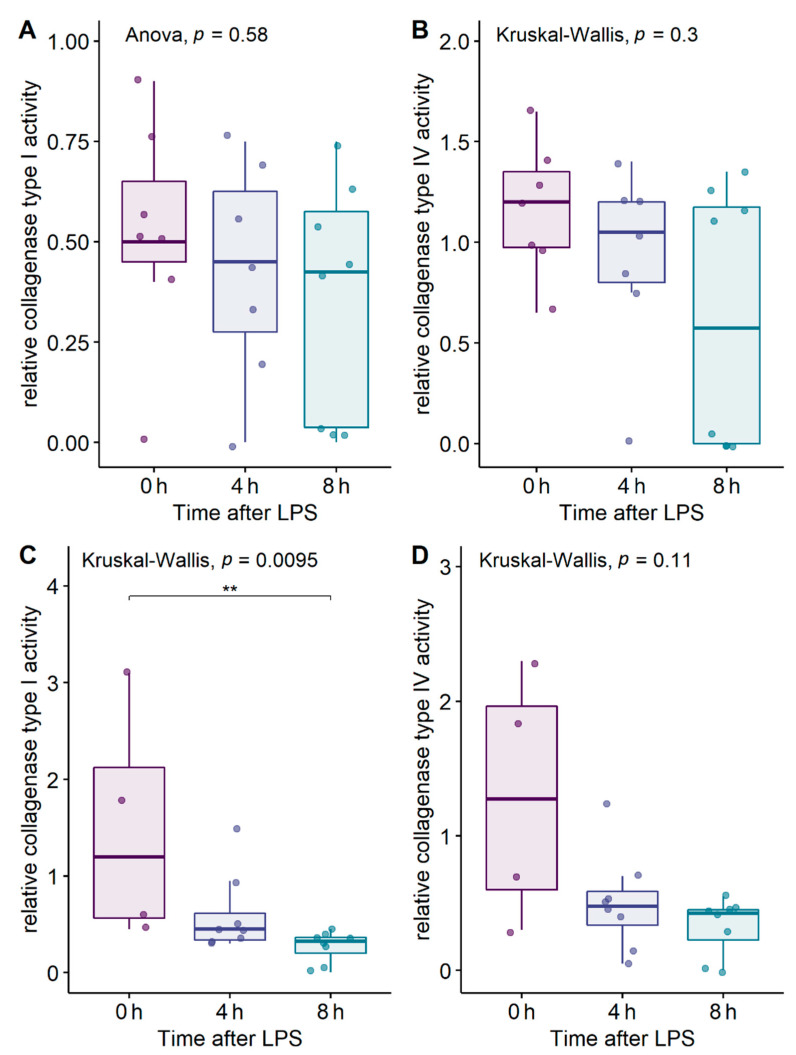
Matrix degrading activity in the jejunum (**A**,**B**) or the ileum (**C**,**D**) measured at 0 h, 4 h or 8 h after the IP LPS challenge. ** *p* < 0.01.

We next wanted to explore whether the LPS-induced shifts in the microbial composition could be linked to the inflammation or tissue remodeling. Therefore, the gene expression of multiple host genes was measured in the lung and small intestinal tissue: inflammatory cytokines (IL1β, IL6, TNFα), inducible nitric oxide synthase (iNOS; associated with oxidative stress) and matrix metalloproteinases (MMP2, MMP7, MMP9 and MMP13) (Figure A1 and Figure A2). Additionally, the collagenase activity towards the collagen type I and IV was measured in the lung and small intestinal tissue (Figure A3). In the lungs, the collagenase activity was below the detection limit of the assay. As the LPS challenge had only a minor effect on the microbiome in the colon, no further analyses were performed on the colon tissue. The associations between the microbial community structure in the lung or small intestine and the host gene expression or the collagenase activity measurements were identified using PERMANOVA (Table 4). No link between the community structure and host measurements could be observed in the ileum. However, the jejunal microbial community structure was significantly associated with both the collagen type I degrading activity and the MMP2 gene expression in the jejunal tissue (Figure 2B and Table 4). Additionally, the jejunal IL6 mRNA expression tended to vary with the community structure (Figure 2B and Table 4). In the lungs, both the MMP2 and MMP9 gene expressions (which are involved in airway remodeling) tended to be associated with the microbial community structure (Figure 2B and Table 4).

As the alpha and beta diversity of both the respiratory and intestinal communities were significantly linked to each other, we further investigated whether the host measurements that were linked to the variations in the beta diversity could also be linked to the alpha diversity. In the jejunum, the host collagenase type I activity could not be linked to the alpha diversity (Chao1 or Shannon), but the MMP2 gene expression tended to be correlated with the jejunal Shannon diversity (R = 0.4; *p* = 0.097). Furthermore, the MMP9 gene expression in the lung tissue was correlated with the microbial Shannon diversity (Figure 2C), but not with the microbial richness (Chao1: R = -0.048; *p* = 0.84). No link between the MMP2 gene expression and the microbial alpha diversity in the lungs was observed (Chao1: R = −0.12; *p* = 0.60—Shannon: R = −0.17; *p* = 0.46). Together, this indicates that the host measurements are more tightly linked to an overall variation in the community structure than to a change in microbial alpha diversity.

**Figure 4:**
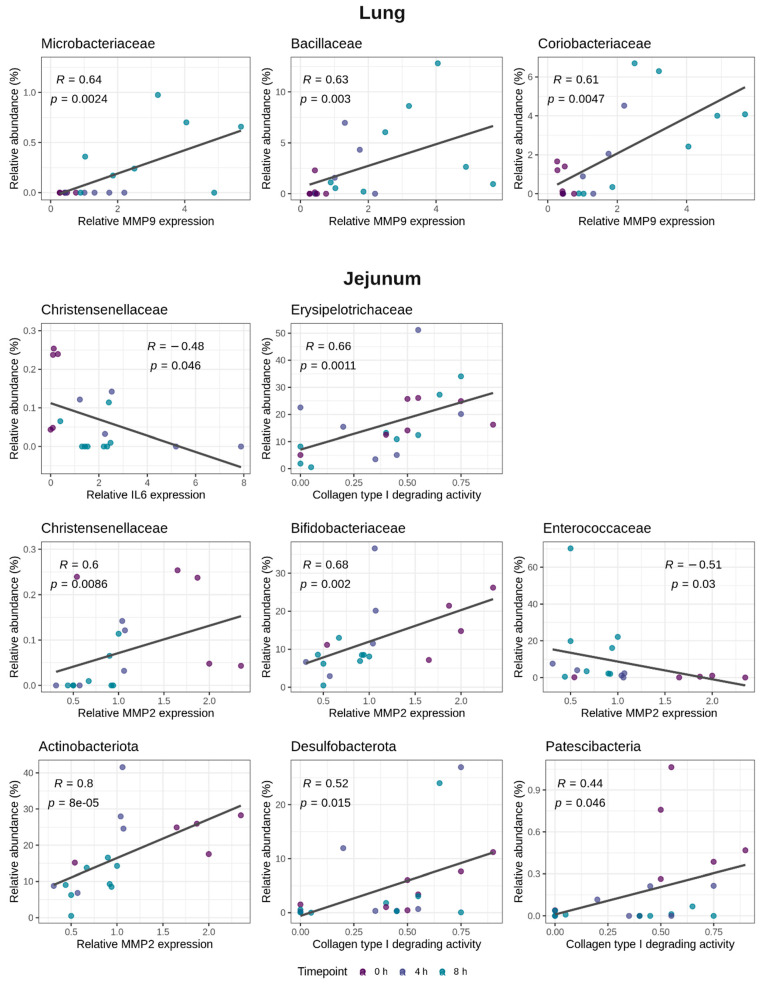
Bacterial taxa in the lung and jejunal microbiome that are related to the host MMP9 mRNA expression in the lungs or the host IL6 mRNA expression, the MMP2 mRNA expression or the collagen type I degrading activity in the jejunum. MaAsLin2 (microbiome multivariable association with linear models) was used to identify the bacterial taxa that were associated with the respective host parameters. Significant associations were confirmed using the Spearman correlation (Spearman R and *p*-values added on the figure).

A MaAsLin2 (microbiome multivariable association with linear models) analysis was performed to further elucidate which specific bacterial taxa within the lung or the jejunal microbiome were associated with either the MMP2 or MMP9 gene expression profiles in the lung, or the jejunal collagen type I degrading activity, the MMP2 or IL6 gene expression. The significant associations detected by the MaAsLin2 were confirmed using the Spearman correlation. In the lungs, the MMP9 gene expression was associated with the families *Microbacteriaceae*, *Bacillaceae* and *Coriobacteriaceae* (Figure 4). No link between the MMP2 gene expression in the lung tissue and the specific bacterial taxa was observed. In the jejunum, the family *Christensenellaceae* was linked to a reduction in the IL6 gene expression (Figure 4). Moreover, also in the jejunum, the phylum Actinobacteriota and the families *Bifidobacteriaceae* and *Christensenellaceae* were associated with an increase in the MMP2 gene expression, whereas the family *Enterococcaceae* was negatively correlated with the MMP2 gene expression (Figure 4). Furthermore, the collagen type I degrading activity in the jejunal tissue was positively associated with the family *Erysipelotrichaceae* and the phyla Desulfobacterota and Patescibacteria (Figure 4).

## 3. Discussion

Systemic inflammatory response syndrome (SIRS) is a severe condition characterized by systemic inflammation, which might lead to multiple organ failure and shock. Various clinical studies have reported gut dysbiosis in SIRS patients. However, the dynamic changes of the microbiome during the early phase of acute SIRS have not been thoroughly investigated. In the current study, we showed that a LPS-induced SIRS rapidly resulted in a small intestinal dysbiosis, which gradually diverged from the healthy microbiome as the endotoxic shock progressed. However, no effect on the large intestinal microbiome was observed. Previous research showed that the fecal microbiome of hamsters was not altered by a systemic LPS challenge, indicating that the microbiome of the large intestine might be less susceptible to a LPS-induced inflammation, at least in the early phase of SIRS [20]. To the best of our knowledge, this is the first study to characterize the response of the small intestinal microbiome to acute SIRS. This small intestinal dysbiosis was characterized by a bloom of *Enterococcaceae* and *Enterobacteriaceae*. Such an expansion of the aerotolerant bacteria is commonly observed in diseases involving inflammation in the gut, such as, amongst others, inflammatory bowel disease, colorectal cancer or food allergies, and might be a reaction to the increased oxygen levels in the inflamed gut [34]. Moreover, the observed expansion of the genus *Escherichia-Shigella* can provide a source of additional LPS, which might further exacerbate the systemic inflammation and so create a positive feedback of dysbiosis and sepsis [28]. Additionally, the LPS-challenge induced a remarkable expansion of the potentially pathogenic genus *Enterococcus* in the small intestine, a genus that was previously reported to be increased in SIRS patients [28,35]. No link between ileal dysbiosis and specific host features could be observed. This is in contrast to the situation in the jejunum, where the endotoxemia-induced microbiome variations could be linked to both the inflammatory cytokine gene expression levels as wells as the factors involved in tissue remodeling. For example, animals with a higher IL6 gene expression had a lower abundance of the butyrate-producing family *Christensenellaceae*. Other bacterial groups were linked to matrix remodeling, such as a higher abundance of the family *Erysipelotrichaceae* in animals with more collagen type I degrading activity in the jejunal tissue. An increase in *Erysipelotrichaceae* in collagen-induced arthritis was previously reported, indicating a possible role in collagen destruction [36]. However, further research is needed to elucidate the functional relevance of these associations.

The lungs are one of the first affected and most vulnerable organs during SIRS and sepsis. Therefore, we further characterized the response of the respiratory microbiome on acute SIRS. In agreement with the dysbiosis in the small intestine and also in the lungs, a marked dysbiosis could be observed due to the LPS-induced inflammation. However, the shift in the respiratory microbiome occurred later in the disease progression. Indeed, at 4 h after the LPS, the respiratory microbiome showed only marginal differences with the healthy lung microbiome, whereas at the 8 h timepoint a clear dysbiosis was observed. When focusing on the lung tissue, an acute lung injury was more pronounced at 8 h after the LPS, as characterized by the alveolar hemorrhages and the induction of the MMP9 gene expression, which is important for tissue remodeling and the disruption of alveo-capillary structures [10,37,38]. Under normal conditions, only limited nutrients are available in the lungs, thereby limiting bacterial growth [23]. However, the SIRS-associated pathological changes result in a higher nutrient availability, leading to the induction of respiratory dysbiosis. In the current study, the systemic inflammation and associated alveolar hemorrhages induced a significant increase in the potentially pathogenic *Pasteurellaceae* and *Streptococcaceae* in the lungs. Although the LPS-induced SIRS resulted in the increase of identical genera in the gut and lungs, no evidence for a bacterial translocation from the gut to the lungs was observed. Indeed, investigation of the specific bacterial sequences present in the gut and lung revealed that different strains were present in either organ, indicating the absence of a gut-lung translocation during the early endotoxemia. The presence of the gut-related bacteria in the lungs of mice after an experimental sepsis and endotoxemia has previously been reported [22]. These authors concluded that these bacteria were originating from the gut. However, although the lung microbial communities were more similar to the gut in post-sepsis lungs, no proof of identical bacteria in the gut and lungs is reported [22]. Therefore, the occurrence of a gut-lung translocation in sepsis and endotoxemia remains elusive.

The overall shift in the respiratory community composition could be associated with both the MMP2 and MMP9 gene expressions in the lung tissue, two MMPs that are well known to be involved in tissue remodeling following an acute lung injury [10,39]. Furthermore, mice with a higher respiratory microbial diversity exhibited greater MMP9 gene expression levels in the lungs. A significant association between the respiratory microbiome and the MMP9 gene expression in the lung has previously been observed in pneumonia patients [40]. Furthermore, a link between the respiratory microbiome and the TNFα levels in both pneumonia and ARDS patients has been reported [22,40]. In our study, no association between the TNFα gene expression levels and variations in the lung microbiome could be observed. TNFα is an important mediator of the epithelial permeability and further research should elucidate whether the respiratory microbiome can be linked to TNFα later in the pathogenesis, when a bacterial translocation occurs.

In conclusion, an acute LPS-induced SIRS had no effect on the colonic microbiome, but induced a rapid dysbiosis of the small intestine, which resembles the microbiome alterations commonly observed in SIRS patients. Later in the disease progression, a dysbiosis of the respiratory microbiome was observed, which seemed to be linked with the MMP9 gene expression levels in the lungs. Although similar bacteria were increased in both the lung and the small intestine, no evidence for a gut-lung translocation was observed. This research provides an important fundamental understanding on the dynamic changes of both the intestinal and respiratory microbiome during the early phases of acute systemic inflammation, and opens opportunities for further research investigating the host-microbiome interactions in the early phases of SIRS.

## 4. Materials and Methods

### 4.1. LPS Challenge Model

Eight-week old female C57BL/6J mice were purchased from Envigo Research Models and Services (C56Bl/6JOlaHsd, Venray, Netherlands). The animals were housed in groups of six mice, in sterile, ventilated cages enriched with rodent homes and nesting papers. The animals had *ad libitum* access to water and food, and acclimatized for 10 days prior to the start of the experiment. In total, 24 mice (four cages of six mice) were included in the experiment. From each cage, four mice were injected intraperitoneally with LPS from *Salmonella enterica serotype abortus equi* (Sigma-Aldrich, Diegem, Belgium) at 200 µg per 20 g bodyweight, which was previously determined to be the LD_100_ dose for C57BL/6J mice. The rectal temperature was measured every two hours after the challenge. From each cage, two mice were euthanized at 4 h after the LPS, and two mice at 8 h after the LPS, after which lung and intestinal tissue samples, as well as intestinal content were collected. The other two mice per cage were used unchallenged (0 h after the LPS timepoint).

### 4.2. Real Time qPCR

The lung and intestinal samples were stored in RNAlater (Ambion, Ghent, Belgium) and RNA was extracted using the Aurum kit (Bio-Rad, Nazareth-Eke, Belgium). cDNA was synthesized using the iScript cDNA synthesis Kit (Bio-Rad) and real time qPCR was performed on the CFX384 detection system (Bio-Rad), using the SensiMix SYBR No-ROX mix (Bioline, Kampenhout, Belgium). The expression levels were normalized to the most stable housekeeping genes, which were determined for each organ using the geNorm Housekeeping Gene Selection Software within qBASE+ (Biogazelle, Zwijnaarde, Belgium) [41]. The primer sequences are listed in Table 5.

### 4.3. Matrix Degradation Assay

The small intestinal tissue (jejunum or ileum) or lung tissue samples were homogenized in a 1% NP-40 buffer as previously described [43]. The protein concentration was measured using the BCA protein assay (Thermo Fisher Scientific, Merelbeke, Belgium) and samples were stored at −20 °C until further analysis. The collagen type I or collagen type IV degradation by the enzymes present in the lung tissue or small intestinal tissues (jejunum or ileum) was assessed using the EnzChek^®^ Gelatinase/Collagenase Assay Kit (Molecular probes, Thermo Fisher Scientific). Duplicate measurements were performed in 200 μL reaction volume containing 20 µL of either a fluorescein labelled substrate (DQ Collagen I (25 μg/mL, D12060) or DQ Collagen IV (25 μg/mL, D12052), 100 μL of the tissue lysate (500 µg/mL) and 80 μL of a reaction buffer (0.5 M Tris–HCl, 1.5 M NaCl, 50 mM CaCl2 and 2 mM sodium azide, pH 7.6). Collagenolytic activity was detected using a fluorometer (excitation 485 nm, emission 527 nm; Fluoroskan Ascent Fluorometer, Thermo Fisher Scientific Inc., Merelbeke, Belgium).

### 4.4. Histopathology of the Lung Sections

The lung tissue was fixed with paraformaldehyde, embedded in paraffin and sectioned at 4 µm. To analyze the lung histology, the samples were stained with hematoxylin and eosin. The degree of lung damage was evaluated on the entire organ sections by a trained pathologist. The lung damage was scored by the presence/absence of neutrophils adhering to the vascular walls, the presence/absence of alveolar hemorrhages and the presence/absence of congested blood vessels in the lungs.

### 4.5. Microbial DNA Isolation, 16S rRNA DNA Sequencing and Bioinformatics

The DNA was extracted from the intestinal digesta (jejunum, ileum or colon samples) or lung tissue using the CTAB method, as previously described by Griffiths et al., with modifications described by Aguirre et al. [43,44]. The bacterial barcoding was performed with a 2-step amplification process using the primers S-D-Bact-0341-b-S-17 (5′-CCTACGGGNGGCWGCAG-3′) and S-D-Bact-0785-a-A-21 (5′-GACTACHVGGGTATCTAATCC-3′) which amplify the V3-V4 region of the 16S rRNA gene as described before [43,45]. The final barcoded libraries were sequenced on two different runs using the Illumina MiSeq v3 technology (2 × 300 bp, paired-end) by Macrogen. The demultiplexing of the amplicon dataset and the deletion of the barcodes was carried out by the sequencing provider. The optimal trimming parameters were determined using the python-based application FIGARO [46]. All further processing was performed in R (v4.1.2) [47]. The raw sequence reads were trimmed, quality-filtered and dereplicated using the *DADA2* algorithm (v1.14.0) [48]. An initial amplicon sequence variant (ASV) table was constructed before the chimaeras were identified using the *removeBimeraDenovo* function. Finally, the taxonomy was assigned using *DADA2*′s native naïve Bayesian classifier against the Silva database (v138) [49]. To construct a phylogenetic tree, the multiple sequence alignment was performed using the *DECIPHER* (v2.14.0) algorithm [50], after which a neighbor-joining tree was constructed using *PHANGORN* (v2.7.0) [51]. This neighbor-joining tree was used as the starting point to fit the final GTR+G+I (generalized time-reversible with gamma rate variation) maximum likelihood tree. The resulting phylogenetic tree and the ASV table were loaded into *Phyloseq* (v1.28.0) [52], after which the potential contaminant chloroplastic and mitochondrial ASVs were removed from the data set. The potential contaminant DNA reads originating from the DNA extraction or the library preparation buffers were identified based on both the DNA concentration and the prevalence of the ASVs in the negative control samples using *decontam* (v1.14.0) [53] and removed from the final dataset.

### 4.6. Statistical Analysis

All statistical analyses were performed in R (v4.1.2) [47]. The effect of the LPS challenge on the body temperature of the mice was assessed using a linear model with “MouseID” as a random factor. The effect of the LPS challenge on the lung histology, host gene expression, small intestinal collagenase activity or microbial alpha diversity was assessed using either ANOVA or Kruskal–Wallis, as appropriate.

Given the coprophagic behavior of the co-housed mice, the cage ID was recorded for each animal and included as a potential confounder into the multivariable microbiome analysis. The microbial alpha diversity (Chao1 richness estimator and the Shannon diversity index) was calculated using *phyloseq*. Prior to the beta diversity analysis, the 16S sequencing data was transformed to portions. The Bray–Curtis distance was used as a measure for the microbial beta diversity. The sample distribution was visualized via nonmetric multidimensional scaling (NMDS) plots. The dispersion (variance) in the beta diversity was calculated using the *betadisper* function in the *vegan* package [54]. The differences in the variances between the groups were tested using ANOVA, followed by a Tukey’s multiple comparison test. The significant differences in the community composition between the groups were determined through a permutational multivariate analysis of variance using distance matrices (PERMANOVA), using the *adonis* function in *vegan*. In case a significant effect of the LPS challenge was observed, the pairwise comparison between the group levels was performed using the function *pairwise.perm.manova* from the *RVAideMemoire* package and the Bonferroni corrected *p*-values were reported [55]. Additionally, to ensure there was no confounding by the observed dispersion effects, the W_d_*-test, which tests the differences in the overall microbiome composition while accounting for the differences in the group dispersion, was used [56]. The differentially abundant taxa (phyla, families or genera) in the intestinal or respiratory microbiome at the different timepoints after the LPS challenge were identified by applying *DESeq2* on the non-rarefied community composition data [57]. The significant differences were obtained using a Wald test followed by a Benjamini–Hochberg multiple hypothesis correction.

The significant associations between the beta diversity and the host measurements (gene expression or collagenase activity data) were identified using PERMANOVA. The associations between the alpha diversity and the host measurements were identified using the Spearman correlation. The identification of the specific bacterial taxa (phylum or family level) that were associated with the host measurements was performed using MaAsLin2 (microbiome multivariable association with linear models) [58], and confirmed using Spearman correlation.

## Figures and Tables

**Table 1:** Alpha diversity of the lung, jejunal, ileal and colonic microbial communities from mice 0 h (unchallenged), 4 h or 8 h after the intraperitoneal LPS injection.

	0 h	4 h	8 h	Global *p*-Value	0 h vs. 4 h	0 h vs. 8 h	4 h vs. 8 h
	mean ± SD				Adjusted *p*-Value		
**Lung**							
Chao1	69.71 ± 43.88	37.5 ± 11.51	78.00 ± 41.10	0.045 *	0.274	0.368	0.040 *
Shannon	3.13 ± 0.32	3.05 ± 0.49	3.22 ± 0.87	0.210	0.843	0.342	0.314
**Jejunum**							
Chao1	79.29 ± 11.25	74.71 ± 35.21	64.00 ± 40.38	0.300	0.862	0.361	0.862
Shannon	2.73 ± 0.22	2.62 ± 0.37	2.18 ± 0.73	0.067 °	0.564	0.078 °	0.206
**Ileum**							
Chao1	61.86 ± 29.99	106.29 ± 84.42	46.00 ± 33.41	0.270	0.684	0.684	0.319
Shannon	2.74 ± 0.23	3.09 ± 0.55	2.15 ± 0.57	0.023 *	0.320	0.155	0.020 *
**Colon**							
Chao1	280.07 ± 40.21	289.00 ± 42.83	286.58 ± 64.43	0.910	1	1	1
Shannon	4.34 ± 0.25	4.50 ± 0.27	4.48 ± 0.19	0.470	0.843	0.843	0.937

* Significant differences between the different timepoints after the intraperitoneal LPS injection (*p* < 0.05). ° Variables that showed a tendency (*p* < 0.1).

**Table 2:** Effect of the intraperitoneal LPS injection on the community variance and community structure in the lung, jejunum, ileum or colon. Beta diversity was assessed using the Bray–Curtis dissimilarity. Community variance was calculated using betadisper and the differences between the groups were tested using ANOVA. Differences in the community structure were tested using the commonly used PERMANOVA test, as well as the W_d_*-test, which is more reliable in case of unequal community dispersions (variances). *Post hoc* analysis results on the significant LPS effects are listed in Table A1.

	Community Variance	Community Structure			
	ANOVA	PERMANOVA		Wd*-Test	
	*p*-Value	R^2^ (%)	*p*-Value	Test-Statistic	*p*-Value
**Lung**	0.836	10.46	0.107	1.11	0.092
**Jejunum**	0.023	15.49	0.018	1.86	0.006
**Ileum**	<0.001	24.09	0.002	2.64	0.001
**Colon**	0.907	10.55	0.435	0.95	0.427

**Table 3:** Differentially abundant families in the lung, jejunal, ileal or colonic microbiota.

Phylum	Class	Order	Family	Mean Abundance (%)			0 h–4 h		0 h–8 h	
				0 h	4 h	8 h	L2FC	p_adj_	L2FC	p_adj_
**Lung**										
Firmicutes	Bacilli	Bacillales	*Bacillaceae*	0.35	2.16	4.12	6.93	0.052	7.58	0.004
Firmicutes	Bacilli	Lactobacillales	*Streptococcaceae*	1.16	1.96	12.77	1.80	0.932	6.28	0.050
Proteobacteria	Gammaproteobacteria	Enterobacterales	*Pasteurellaceae*	0.00	0.78	11.02	8.47	0.058	10.63	0.003
**Jejunum**										
Bacteroidota	Bacteroidia	Bacteroidales	*Muribaculaceae*	0.10	2.70	0.54	5.67	0.002	3.78	0.071
Bacteroidota	Bacteroidia	Bacteroidales	*Prevotellaceae*	0.00	0.09	0.06	8.57	0.036	7.74	0.115
Bacteroidota	Bacteroidia	Cytophagales	*Spirosomaceae*	0.00	0.28	0.05	10.09	0.003	8.23	0.044
Firmicutes	Bacilli	Lactobacillales	*Enterococcaceae*	0.26	2.28	17.06	2.05	0.183	4.65	0.003
Firmicutes	Clostridia	Oscillospirales	*Ruminococcaceae*	0.03	0.49	1.07	5.41	0.030	3.44	0.263
Proteobacteria	Alphaproteobacteria	Sphingomonadales	*Sphingomonadaceae*	0.00	0.29	0.02	23.84	0.000	20.32	0.000
Proteobacteria	Gammaproteobacteria	Burkholderiales	*Sutterellaceae*	0.00	0.07	0.11	7.27	0.003	7.67	0.003
Proteobacteria	Gammaproteobacteria	Enterobacterales	*Enterobacteriaceae*	0.01	7.35	0.03	9.78	0.032	1.84	0.857
Proteobacteria	Gammaproteobacteria	Enterobacterales	*Yersiniaceae*	0.00	0.06	0.04	8.26	0.032	8.69	0.044
Proteobacteria	Gammaproteobacteria	Pseudomonadales	*Pseudomonadaceae*	0.00	0.32	0.04	7.68	0.032	4.53	0.324
**Ileum**										
Actinobacteriota	Coriobacteriia	Coriobacteriales	*Eggerthellaceae*	4.50	0.76	0.11	-2.22	0.216	-5.24	0.000
Bacteroidota	Bacteroidia	Bacteroidales	*Marinifilaceae*	0.00	0.15	0.03	7.60	0.021	5.21	0.113
Bacteroidota	Bacteroidia	Bacteroidales	*Muribaculaceae*	2.42	21.50	3.93	4.43	0.003	1.48	0.343
Bacteroidota	Bacteroidia	Bacteroidales	*Prevotellaceae*	0.00	0.21	0.01	6.56	0.007	3.01	0.240
Firmicutes	Bacilli	Lactobacillales	*Enterococcaceae*	1.00	9.76	9.17	4.33	0.016	6.20	0.000
Firmicutes	Bacilli	Lactobacillales	*Streptococcaceae*	0.03	0.19	0.33	3.39	0.070	5.71	0.000
Firmicutes	Clostridia	Christensenellales	*Christensenellaceae*	0.12	0.01	0.00	-2.46	0.377	-4.42	0.048
Firmicutes	Clostridia	Clostridiales	*Clostridiaceae*	2.09	4.56	4.57	2.04	0.182	3.23	0.008
Proteobacteria	Alphaproteobacteria	Caulobacterales	*Caulobacteraceae*	0.00	0.08	0.93	7.21	0.039	10.53	0.001
Proteobacteria	Alphaproteobacteria	Rhizobiales	*Beijerinckiaceae*	0.00	1.52	9.32	10.58	0.017	11.32	0.008
Proteobacteria	Alphaproteobacteria	Rhizobiales	*Rhizobiaceae*	0.00	1.64	4.98	10.69	0.000	11.63	0.000
Proteobacteria	Alphaproteobacteria	Sphingomonadales	*Sphingomonadaceae*	0.00	1.03	2.76	24.62	0.000	26.24	0.000
Proteobacteria	Gammaproteobacteria	Burkholderiales	*Burkholderiaceae*	0.00	0.00	9.75	0.00	1.000	30.00	0.000
Proteobacteria	Gammaproteobacteria	Enterobacterales	*Enterobacteriaceae*	0.05	0.20	10.67	2.65	0.796	9.43	0.014
Proteobacteria	Gammaproteobacteria	Enterobacterales	*Pasteurellaceae*	0.00	0.00	6.91	0.00	1.000	30.00	0.000
**Colon**										
Deferribacterota	Deferribacteres	Deferribacterales	*Deferribacteraceae*	0.00	0.13	0.33	4.89	0.267	8.30	0.000

Significant differences in the family level abundance in the lung, jejunal, ileal or colonic microbiota from mice 4 h or 8 h after the intraperitoneal LPS challenge, as compared to the unchallenged mice. The taxonomic classification and the log2 fold change (L2FC) of the DESeq2-normalized abundance of each family are shown. Positive L2FC values indicate an increase in the abundance of the respective family after the LPS challenge, while the negative values indicate a decrease.

**Table 4:** Relationship of the microbial alpha diversity, host gene expression measurements or small intestinal collagenase activity measurements with the bacterial community composition. Beta diversity was assessed using the Bray–Curtis dissimilarity. Associations of the measured variables with the beta diversity were tested using PERMANOVA.

	Lung		Jejunum		Ileum		Colon	
	R^2^ (%)	*p*-Value	R^2^ (%)	*p*-Value	R^2^ (%)	*p*-Value	R^2^ (%)	*p*-Value
***Microbial alpha diversity***								
Chao1	6.00	0.059 °	9.70	0.003 **	4.98	0.440	7.93	0.029 *
Shannon	5.97	0.055 °	11.14	0.002 **	13.72	0.003 **	13.42	0.001 ***
***Host gene expression***								
IL1β	4.68	0.787	5.09	0.461	-	-	-	-
IL6	4.99	0.641	7.94	0.099 °	5.31	0.625	-	-
iNOS	5.77	0.324	4.32	0.544	6.13	0.409	-	-
TNFα	4.67	0.795	4.45	0.501	4.14	0.803	-	-
MMP2	6.69	0.086 °	9.32	0.045 *	5.44	0.520	-	-
MMP7	-	-	7.16	0.255	1.21	1.000	-	-
MMP9	6.73	0.088 °	7.77	0.155	6.63	0.320	-	-
MMP13	5.49	0.451	6.24	0.298	6.22	0.404	-	-
***Host collagenase activity***								
Type I	-	-	8.69	0.031 *	8.97	0.103	-	-
Type IV	-	-	3.88	0.639	7.58	0.206	-	-

Significant associations are highlighted with: * *p* < 0.05, ** *p* < 0.01 or *** *p* < 0.001. Variables that tended to vary with the Bray–Curtis beta diversity are highlighted using ° *p* < 0.1. “-” indicates that the host measurements were lacking.

**Table 5:** Primer sequences used for qPCR analysis.

Gene Name	Forward Primer	Reverse Primer	Reference
***IL1β***	CACCTCACAAGCAGAGCACAAG	GCATTAGAAACAGTCCAGCCCATAC	[42]
***IL6***	TAGTCCTTCCTACCCCAATTTCC	TTGGTCCTTAGCCACTCCTTC	[11]
***TNFα***	ACCCTGGTATGAGCCCATATAC	ACACCCATTCCCTTCACAGAG	[11]
***iNOS***	TGGTCCGCAAGAGAGTGCT	CCTCATTGGCCAGCTGCTT	[11]
***MMP2***	AGATCTTCTTCTTCAAGGACCGGTT	GGCTGGTCAGTGGCTTGGGGTA	[42]
***MMP7***	ACTTCAGACTTACCTCGGATCG	TCCCCCAACTAACCCTCTTGA	[11]
***MMP9***	CTGGACAGCCAGACACTAAAG	CTCGCGGCAAGTCTTCAGAG	[42]
***MMP13***	TTTATTGTTGCTGCCCATGA	GGTCCTTGGAGTGATCCAGA	[42]
***β-actin***	GCTTCTAGGCGGACTGTTACTGA	GCCATGCCAATGTTGTCTCTTAT	[42]
***β2M***	ATGCACGCAGAAAGAAATAGCAA	AGCTATCTAGGATATTTCCAATTTTTGAA	[42]
***GADPH***	TGAAGCAGGCATCTGAGGG	CGAAGGTGGAAGAGTGGGAG	[11]
***Rpl***	CCTGCTGCTCTCAAGGTT	TGGCTGTCACTGCCTGGTACTT	[11]
***Ubc***	AGGTCAAACAGGAAGACAGACGTA	TCACACCCAAGAACAAGCACA	[11]
***Ywhaz***	GCAACGATGTACTGTCTCTTTTGG	GTCCACAATTCCTTTCTTGTCATC	[42]

## Data Availability

The raw sequencing data and corresponding metadata are available on NCBI SRA under the BioProject PRJNA884184.

## Appendix A

**Table A1:** Statistical results on the *post hoc* analysis of the LPS effect (0 h, 4 h or 8 h after the LPS) on the microbial community variance and community structure in the lungs or intestine (jejunum, ileum or colon). Beta diversity was assessed using the Bray–Curtis dissimilarities. Community variances were calculated using *betadisper* and tested using ANOVA, whereas the differences in the community structure were tested using PERMANOVA (Table 2). *Post hoc* analysis on the LPS effect on the community variance was performed using Tukey’s multiple comparison of the means. *Post hoc* analysis on the LPS effect on the community structure was performed using the pairwise PERMANOVA, resulting in the Bonferroni corrected adjusted *p*-values.

	Community Variance	Community Structure	
		PERMANOVA	Wd*-Test
	Adjusted *p*-Value		
**Lung**			
**0 h vs. 4 h**	0.817	1	1
**0 h vs. 8 h**	0.981	0.140	0.138
**4 h vs. 8 h**	0.900	0.014	0.024
**Jejunum**			
**0 h vs. 4 h**	0.046	0.321	0.315
**0 h vs. 8 h**	0.035	0.025	0.015
**4 h vs. 8 h**	1	0.853	0.759
**Ileum**			
**0 h vs. 4 h**	0.020	0.023	0.033
**0 h vs. 8 h**	<0.001	0.002	0.003
**4 h vs. 8 h**	0.037	0.082	0.120
**Colon**			
**0 h vs. 4 h**	0.988	0.469	0.429
**0 h vs. 8 h**	0.953	0.670	0.636
**4 h vs. 8 h**	0.901	1	1

**Table A2:** Differentially abundant genera in the lung, jejunum, ileum or colon of mice after the intraperitoneal LPS challenge.

Phylum	Class	Family	Genus	Mean Abundance (%)			0 h–4 h		0 h–8 h	
				0 h	4 h	8 h	L2FC	p_adj_	L2FC	p_adj_
**Lung**										
Bacteroidota	Bacteroidia	*Tannerellaceae*	*Parabacteroides*	0.28	0.00	0.08	−24.33	0.000	−9.25	0.153
Firmicutes	Bacilli	*Bacillaceae*	*Pseudogracilibacillus*	0.00	0.74	1.25	21.65	0.000	25.95	0.000
Firmicutes	Bacilli	*Planococcaceae*	*Sporosarcina*	0.01	3.53	1.35	10.54	0.005	10.43	0.005
Firmicutes	Bacilli	*Erysipelatoclostridiaceae*	*Erysipelatoclostridium*	0.41	0.00	1.93	−23.47	0.000	0.38	0.979
Firmicutes	Bacilli	*Erysipelatoclostridiaceae*	*Erysipelotrichaceae UCG-003*	0.24	0.00	0.12	−22.46	0.000	−24.26	0.000
Firmicutes	Bacilli	*Streptococcaceae*	*Streptococcus*	1.16	1.86	12.77	1.57	0.978	6.90	0.017
Firmicutes	Clostridia	*Oscillospiraceae*	*Oscillospira*	0.15	0.00	0.00	−23.38	0.000	−25.19	0.000
Firmicutes	Clostridia	*Oscillospiraceae*	*UCG-005*	0.07	0.00	0.04	−19.90	0.000	−4.05	0.629
Proteobacteria	Gammaproteobacteria	*Sutterellaceae*	*Parasutterella*	0.76	0.29	0.00	−1.84	0.998	−24.37	0.000
**Jejunum**										
Bacteroidota	Bacteroidia	*Muribaculaceae*	*Family_Muribaculaceae*	0.10	2.70	0.54	5.66	0.038	4.14	0.138
Bacteroidota	Bacteroidia	*Spirosomaceae*	*Flectobacillus*	0.00	0.28	0.05	10.07	0.038	9.84	0.032
Firmicutes	Bacilli	*Erysipelotrichaceae*	*Turicibacter*	1.23	7.75	7.33	3.91	0.221	5.29	0.032
Firmicutes	Bacilli	*Enterococcaceae*	*Enterococcus*	0.26	2.28	17.06	4.19	0.174	9.44	0.000
Firmicutes	Clostridia	*Clostridiaceae*	*Candidatus Arthromitus*	0.00	0.51	0.03	8.16	0.038	4.90	0.326
Proteobacteria	Gammaproteobacteria	*Sutterellaceae*	*Parasutterella*	0.00	0.07	0.09	7.57	0.038	7.99	0.027
**Ileum**										
Actinobacteriota	Coriobacteriia	*Eggerthellaceae*	*Enterorhabdus*	4.12	0.71	0.11	−1.87	0.264	−4.61	0.002
Bacteroidota	Bacteroidia	*Marinifilaceae*	*Odoribacter*	0.00	0.15	0.03	7.79	0.024	5.20	0.131
Bacteroidota	Bacteroidia	*Muribaculaceae*	*Family_Muribaculaceae*	2.27	20.58	3.73	4.82	0.002	1.58	0.335
Bacteroidota	Bacteroidia	*Prevotellaceae*	*Alloprevotella*	0.00	0.11	0.00	6.86	0.043	3.55	0.298
Firmicutes	Bacilli	*Enterococcaceae*	*Enterococcus*	1.00	9.76	9.17	4.42	0.023	6.52	0.000
Firmicutes	Bacilli	*Streptococcaceae*	*Lactococcus*	0.00	0.00	0.14	0.39	1.000	7.59	0.024
Firmicutes	Bacilli	*Streptococcaceae*	*Streptococcus*	0.02	0.19	0.19	4.05	0.071	5.66	0.005
Firmicutes	Clostridia	*Clostridiaceae*	*Candidatus Arthromitus*	0.68	1.12	1.95	2.00	0.264	3.93	0.010
Proteobacteria	Alphaproteobacteria	*Caulobacteraceae*	*Brevundimonas*	0.00	0.08	0.93	7.62	0.037	10.81	0.001
Proteobacteria	Alphaproteobacteria	*Beijerinckiaceae*	*Methylobacterium-Methylorubrum*	0.00	1.52	9.32	10.35	0.025	11.80	0.007
Proteobacteria	Alphaproteobacteria	*Rhizobiaceae*	*Allorhizobium-Neorhizobium-Pararhizobium-Rhizobium*	0.00	1.64	4.98	10.52	0.000	12.17	0.000
Proteobacteria	Alphaproteobacteria	*Sphingomonadaceae*	*Sphingomonas*	0.00	0.88	2.74	24.27	0.000	26.77	0.000
Proteobacteria	Gammaproteobacteria	*Burkholderiaceae*	*Ralstonia*	0.00	0.00	9.75	0.00	1.000	30.00	0.000
Proteobacteria	Gammaproteobacteria	*Enterobacteriaceae*	*Escherichia-Shigella*	0.00	0.08	10.67	20.11	0.000	28.76	0.000
Proteobacteria	Gammaproteobacteria	*Pasteurellaceae*	*Muribacter*	0.00	0.00	6.91	0.00	1.000	30.00	0.000
**Colon**										
Deferribacterota	Deferribacteres	*Deferribacteraceae*	*Mucispirillum*	0.00	0.13	0.33	5.02	0.207	8.33	0.000
Desulfobacterota	Desulfovibrionia	*Desulfovibrionaceae*	*Bilophila*	0.00	0.07	0.10	5.25	0.012	5.90	0.002
Firmicutes	Clostridia	*Lachnospiraceae*	*[Eubacterium] xylanophilum group*	0.65	0.33	0.13	−0.93	0.798	−2.30	0.047
Firmicutes	Clostridia	*Ruminococcaceae*	*[Eubacterium] siraeum group*	0.04	0.10	0.25	1.64	0.551	2.93	0.038

Significant differences in the genus level abundance in the lung, jejunal, ileal or colonic microbiota from mice 4 h or 8 h after the intraperitoneal LPS challenge as compared to the unchallenged mice (0 h) The taxonomic classification and the log2 fold change (L2FC) of the DESeq2-normalized abundance of each genus are shown. Positive L2FC values indicate an increase in the abundance of the respective genus after the LPS challenge, while the negative values indicate a decrease.
